# Signals from the Circle: Tricarboxylic Acid Cycle Intermediates as Myometabokines

**DOI:** 10.3390/metabo11080474

**Published:** 2021-07-23

**Authors:** Jennifer Maurer, Miriam Hoene, Cora Weigert

**Affiliations:** 1 Department for Diagnostic Laboratory Medicine, Institute for Clinical Chemistry and Pathobiochemistry, University Hospital Tuebingen, 72076 Tuebingen, Germany; jennifer.maurer@med.uni-tuebingen.de (J.M.); miriam.hoene@med.uni-tuebingen.de (M.H.); 2 Institute for Diabetes Research and Metabolic Diseases, Helmholtz Center Munich, University of Tuebingen, 72076 Tuebingen, Germany; 3 German Center for Diabetes Research (DZD), 85764 Oberschleissheim, Germany

**Keywords:** TCA cycle, exercise, myometabokine, exercise adaptation, liver, arterio-venous difference, succinate, citrate

## Abstract

Regular physical activity is an effective strategy to prevent and ameliorate aging-associated diseases. In particular, training increases muscle performance and improves whole-body metabolism. Since exercise affects the whole organism, it has countless health benefits. The systemic effects of exercise can, in part, be explained by communication between the contracting skeletal muscle and other organs and cell types. While small proteins and peptides known as myokines are the most prominent candidates to mediate this tissue cross-talk, recent investigations have paid increasing attention to metabolites. The purpose of this review is to highlight the potential role of tricarboxylic acid (TCA) metabolites as humoral mediators of exercise adaptation processes. We focus on TCA metabolites that are released from human skeletal muscle in response to exercise and provide an overview of their potential auto-, para- or endocrine health-promoting effects.

## 1. Introduction

Regular physical activity has well-known beneficial effects in the prevention and treatment of numerous disorders, including metabolic and cardiovascular diseases, as well as the risk factors obesity, hyperglycemia, insulin resistance and hypertension [1,2]. Metabolic parameters that have been reported to be improved by exercise include glucose homeostasis, insulin sensitivity [3,4], and liver fat [5]. Therefore, physical exercise is often part of the preventive measures and therapeutic schemes used to combat type 2 diabetes (T2D) [6]. In general, most subjects participating in lifestyle interventions benefit from both endurance and resistance training [7,8]. However, there is a broad range in the improvement of fitness and metabolic parameters between individuals that cannot be fully explained to date [9,10]. Thus, our understanding of how the health-promoting effects of exercise are mediated is still incomplete. This is particularly true for the systemic effects of exercise, i.e., the response of and communication between different organs and cell types.

**Figure 1:**
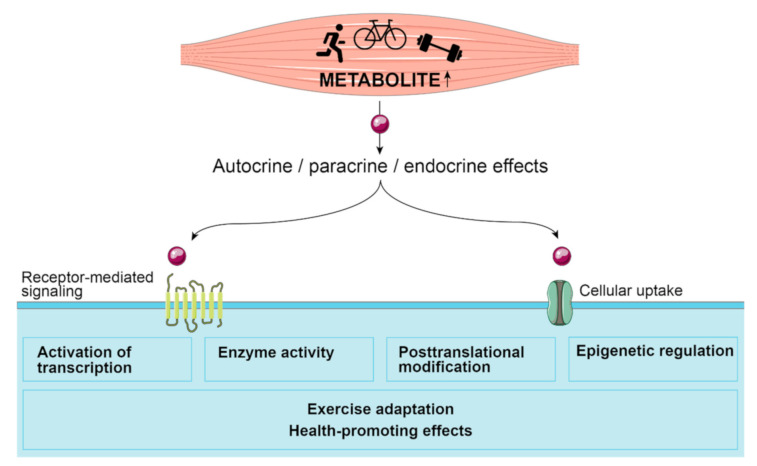
**The concept of myometabokines.** Metabolites released from the contracting skeletal muscle can regulate transcription and translation, enzyme activities and signaling cascades by binding to receptors or after transporter-mediated cellular uptake. By initiating these events in the skeletal muscle or in other tissues, adaptive responses to physical activity are supported. The figure was created using Servier Medical Art templates, which are licensed under a Creative Commons Attribution 3.0 Unported License; https://smart.servier.com (accessed on 22 June 2021).

Skeletal muscle is a key player during physical exercise. It represents the organ with the highest insulin-dependent uptake of glucose in the human body [11] and relies on a well-orchestrated interorgan crosstalk to support the heightened energy demand. In addition to immediate changes in energy metabolism, this signaling network also initializes adaptive processes on a whole-body level. Besides neuromuscular and hormonal mechanisms, myokines have gained attention as humoral mediators of exercise adaptation processes in the last two decades. The term myokine (gr. *mys* = muscle, *kinesis* = motion) was coined in 2003 by Bente Pedersen and colleagues [12] to denote cytokines and other small proteins or peptides that are produced, expressed and released by contracting skeletal muscle fibers and mediate paracrine or endocrine effects [13]. In analogy, we recently introduced the term “myometabokine” [14,15] to denominate metabolites which are released from skeletal muscle in response to acute exercise and can regulate signaling processes involved in exercise adaptation in an auto-, para- or endocrine manner (Figure 1).

Among the metabolites fulfilling these criteria, intermediates of the tricarboxylic acid (TCA) cycle are particularly intriguing myometabokine candidates. Current immunologic and cancer research provided new insights into the signaling properties of TCA cycle metabolites, which often accumulate during the metabolic reprogramming of tumor cells and were, therefore, named oncometabolites [16,17,18]. The growing knowledge of the physiological importance of receptors and transporters of TCA cycle metabolites, and their connection to intracellular signaling pathways and epigenetic modifications, contributes to a picture of TCA metabolites as second messengers, and of the TCA cycle as a signaling hub [19,20,21]. During skeletal muscle contraction, the flux through the TCA cycle is estimated to increase up to 100-fold in order to maintain energy homeostasis by producing sufficient amounts of reducing equivalents [22,23]. As a consequence, TCA cycle intermediates can accumulate and be released by myofibers which, as will be detailed below, have been shown to contribute to the systemic increase in these metabolites during exercise. Thus, TCA intermediates could function as myometabokines signaling from the contracting skeletal muscle cells.

In this narrative review, we provide an overview of the current knowledge and understanding of TCA cycle intermediates as mediators of the adaptive response to physical activity in humans. We report local and systemic changes in their concentrations, as well as tissue fluxes due to acute exercise, and discuss their potential significance as auto-, para- or endocrine messengers.

## 2. The TCA Cycle in the Exercising Skeletal Muscle

The TCA cycle is located in the mitochondrial matrix and connects multiple catabolic and anabolic pathways. In the exercising skeletal muscle, the principal function of the TCA cycle is to oxidize acetyl-CoA derived from the oxidative decarboxylation of pyruvate and from the β-oxidation of fatty acids to generate reducing equivalents (NADH, FADH_2_) for the synthesis of ATP [23,24]. The huge exercise-induced increase in the TCA cycle flux is accompanied by an expansion of the intramuscular TCA intermediate pool, which is greatest within the first minutes after the onset of exercise [23,25,26]. While the acetyl unit does not contribute to the net increase in TCA metabolites, the anaplerotic conversion of pyruvate and glutamate to α-ketoglutarate and alanine via alanine aminotransferase (ALAT) [27,28] exceeds the rate of cataplerosis. In accordance, TCA intermediates of the second span of the cycle, including reactions from α-ketoglutarate to oxaloacetate, contribute most to the increased pool size [23,27]. With prolonged exercise, anaplerosis becomes less dominant, in part due to enhanced amino acid oxidation [24]. The prominent role of the TCA cycle during exercise is also evident by the increase in TCA cycle enzyme activities in the trained skeletal muscle [22].

**Figure 2:**
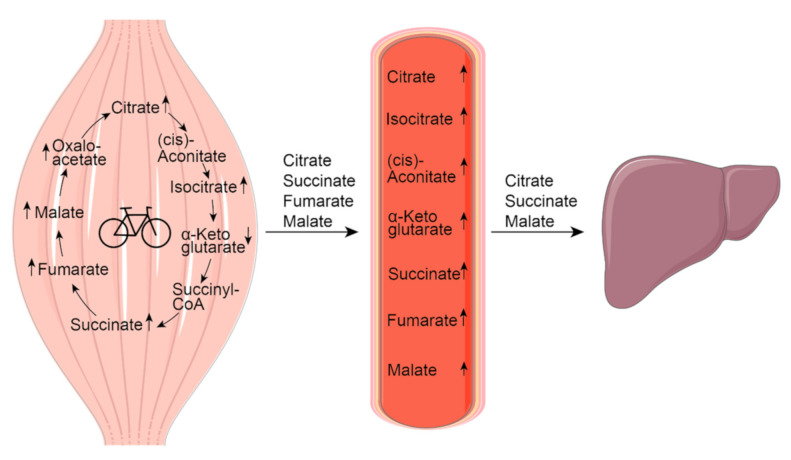
**Exercise-induced changes in TCA intermediate concentrations and fluxes.** Intramuscular and plasma concentrations of most TCA intermediates increase above resting levels in the contracting skeletal muscle [23,27]. There is evidence of an increased muscular release of citrate [35,36], succinate [37,38], fumarate [38] and malate [38,39], as shown by analyses of arterio-venous differences. Citrate, succinate and malate are taken up into the hepato-splanchnic region, suggesting an uptake by the liver [36,39]. The figure was created using Servier Medical Art templates, which are licensed under a Creative Commons Attribution 3.0 Unported License; https://smart.servier.com (accessed on 22 June 2021).

Figure 2 summarizes the regulation of TCA intermediates during exercise. The plasma concentrations of almost all TCA metabolites can be increased after both endurance and resistance exercise [29]. In this review, we focus on citrate, succinate, fumarate and malate, which have been shown to be released from the working muscle during endurance exercise in humans using different approaches (Table 1). The method that provides the best evidence for the release of a metabolite into the circulation is the measurement of arterial-to-venous concentration differences. This procedure has been optimized in a one-legged knee extension protocol, where the other leg can serve as a resting control [30]. Alternatively, microdialysis can be performed to detect an increase in the interstitium [31]. Such a local increase can be equally relevant, since the metabolites may exert auto- or paracrine functions.

We also include α-ketoglutarate, which, together with succinate and fumarate, regulates the enzymatic activity of α-ketoglutarate-dependent dioxygenases (αKGDD)s and has recently been linked to the response to resistance exercise [32]. Isocitrate has been reported to be increased in plasma and interstitial fluid during exercise [33,34], suggesting a muscular release, but there is no evidence from measurements of arterio-venous differences to date. Since we did not find any hints pointing towards a signaling function of isocitrate, it will not be discussed in detail in this review. Few data are available on the plasma concentrations of cis-aconitate and oxaloacetate in exercising humans, and to the best of our knowledge, no validation of their release from skeletal muscle has been reported.

## 3. Citrate

The physiological concentration of citrate in human blood varies between 30 and 400 µM [40]. Exercise induces an increase in the plasma levels by a maximum of two-fold [35,41,42]. Similarly, the intramuscular concentration reaches levels of 1.7-fold over the resting concentration during the first 15 min of exercise, and remains elevated while exercise is continued [23,27]. Further studies using comparable exercise protocols (cycling, 60–75% VO_2_max) measured up to three-fold increases in intramuscular citrate concentrations at later timepoints of ongoing exercise (40, 60 and 75 min) [25,43,44]. A corresponding 1.6-fold increase in citrate in the skeletal muscle interstitial fluid [34] and an elevated release from skeletal muscle in the post-exercise recovery phase [36] have been reported. Citrate is taken up by the hepato-splanchnic bed in the resting state [39], and this uptake was found to be increased during exercise in another study [36] (Figure 2).

Cellular citrate uptake is mediated by the solute carrier (SLC) 13A5 in cotransport with four Na^+^ [45]. The monocarboxylate transporter is selective for citrate over other TCA intermediates and is predominantly expressed in the liver, brain and testis. It shows maximal activity at physiologic pH and has a K_m_ value of ~500 µM [45,46,47,48]. Thus, cellular uptake could be expected to be increased during exercise when blood citrate concentrations are elevated.

Furthermore, the dicarboxylate transporter SLC13A3 that is mainly expressed in liver, brain and kidney is able to cotransport citrate with three Na^+^. Acidic pH stimulates citrate transport via SLC13A3 by shifting the equilibrium from the monocarboxylic towards the dicarboxylic form [45].

Citrate can be transported from the mitochondria into the cytosol in exchange for malate via the mitochondrial citrate carrier SLC25A1, which is expressed in high levels in the liver, pancreas and kidney [49]. Cytosolic citrate is known to act as a fuel-sensing molecule in the Randle cycle by two mechanisms [50]: firstly by inhibiting phosphofructokinase 1 (PFK-1), leading to the downregulation of glycolysis [51], and secondly by allosterically activating acetyl-CoA carboxylase 2 (ACC-2), causing the accumulation of malonyl-CoA which, in turn, inhibits fatty acid oxidation [52,53]. Mice lacking the plasma membrane transporter, SLC13A5, show a phenotype of increased glycolysis, lipid oxidation and mitochondrial biogenesis and reduced fatty acid synthesis that may result from impaired regulation by citrate [54]. The phenotype associated with the mitigated cellular uptake of citrate underlines the relevance of citrate as substrate and activator of hepatic fatty acid biosynthesis. During exercise, when both glycolysis and fatty acid oxidation are increased, this regulatory role appears to be contra-intuitive. However, Ruderman et al. [53] postulated that the exercise-induced transient activation of AMP-activated protein kinase (AMPK) abrogates both basal and citrate-mediated activation of ACC-2, allowing for an increased fatty acid oxidation.

Further signaling properties of citrate are known from studies on its role in immune regulation and tumorigenesis [16,18,55]: cytosolic citrate is cleaved into acetyl-CoA and oxaloacetate through the enzyme ATP-citrate lyase, and can provide the acetyl moiety for histone acetylation, which has been implicated in the activation of glycolytic and proliferative genes [56]. Furthermore, citrate-derived metabolites such as itaconate, oxaloacetate, acetyl-CoA, and fatty acids have been implicated in the regulation of inflammatory cytokine activation and NO and ROS production. Whether exercise-regulated citrate is implicated in the modulation of immune responses and inflammation induced by regular physical activity has not been addressed to date [57].

## 4. α-Ketoglutarate

The plasma levels of α-ketoglutarate average 20 µM in healthy humans [40]. Cycling for 30 min at 80% VO_2_max resulted in 1.5-fold higher plasma levels compared to the resting state [58]. Increased plasma concentrations were also detected after a marathon run [38,41], while no change was measured in a study of 10 min treadmill running until exhaustion [38]. According to a study comparing endurance and resistance exercise, the increase in α-ketoglutarate plasma levels may be more pronounced after resistance exercise [59]. Notably, both intramuscular [23,27] and interstitial concentrations [34] of α-ketoglutarate are decreased during cycling exercise compared to the resting state (Figure 2). Based on these data, it appears unlikely that skeletal muscle contributes to the elevated blood levels of α-ketoglutarate after endurance exercise.

A recent paper [32] suggested α-ketoglutarate as a signaling molecule in mice performing resistance exercise via the cell surface receptor for α-ketoglutarate, OXGR1 (GPR99). They showed that α-ketoglutarate supplementation stimulated thermogenesis and lipolysis via OXGR1 expressed on adrenal glands. While the authors also suggest that α-ketoglutarate may mediate muscle hypertrophy in this setting, further evidence is needed to conclusively show an effect beyond the increase in lean mass.

In addition to its role as an activator of OXGR1, α-ketoglutarate is a substrate for the large family of aKGDDs. After its cellular uptake by transporters of the SLC13A family (both SLC13A2 and SLC13A3 have been reported to transport α-ketoglutarate) [45,46], aKGDDs use α-ketoglutarate as the obligatory co-substrate for oxidation of their substrates, producing carbon dioxide and succinate as by-products. These enzymes are involved in epigenetic regulation by mediating histone and DNA demethylation, as well as in non-epigenetic effects through the stabilization of the transcription factor hypoxia-inducible factor 1 (HIF-1) [21,60,61,62]. Notably, the TCA intermediates succinate and fumarate are inhibitors of aKGDDs. Consequently, changes in the ratio between the co-substrate α-ketoglutarate and the inhibitors succinate and fumarate regulate aKGDD activity [63,64]. As will be detailed below, the reported increase in plasma concentrations of these aKGDD inhibitors exceeds the increase in α-ketoglutarate during exercise, and regulation of the intramuscular concentrations also points towards an elevation of the ratio of succinate and fumarate to α-ketoglutarate. Thus, inhibition of aKGDDs, rather than activation, is a likely event in exercising humans.

## 5. Succinate

The blood concentration of succinate in healthy humans varies between 5 and 30 µM [40]. Both endurance and resistance exercise cause an elevation in plasma concentrations [59]. The increase ranges from 2.5- to 8-fold, depending on the intensity and duration of the exercise bout, and is followed by a rapid decline in the recovery phase [37,38,65,66]. The contracting skeletal muscle releases succinate during exercise [37,39], indicating that skeletal muscle contributes to the increase in plasma succinate. Skeletal muscle biopsies that were taken during an acute ergometer exercise at 70% VO_2_max showed elevated intramuscular succinate concentrations compared to the resting state [27]. The intramuscular content was highest after 5 min of exercise (~4.5-fold) and diminished while exercise was ongoing, but still exceeded resting levels at the end of exercise [27]. This pattern, which mirrors the pronounced increase in lactate [39], suggests that the rapid increase in succinate is caused by the increased flux through glycolysis and substrate-driven conversion of pyruvate to acetyl-CoA by the pyruvate dehydrogenase complex and to α-ketoglutarate via ALAT.

Succinate is generated within the TCA cycle from α-ketoglutarate by a reaction catalyzed by the α-ketoglutarate dehydrogenase complex followed by the succinyl-CoA synthetase-catalyzed conversion of succinyl-CoA. The transport of intramitochondrial and cytosolic succinate occurs via the dicarboxylate carrier SLC25A10 [67]. This facilitates the transport of succinate and malate in exchange for phosphate, sulfate, and thiosulfate. The secretion of succinate from the myofibers into the extracellular space during exercise is mediated by the monocarboxylate transporter 1 (SLC16A1). The intracellular acidification caused by exercise enables the secretion, since a decreased cytosolic pH favors the protonated, monocarboxylic form of succinate [37]. Concordantly, an exercise-induced increase in succinate in the skeletal muscle interstitial fluid has been reported [34,37]. While this supports an auto- and paracrine action of succinate in the working muscle, we recently reported evidence of succinate being taken up into the hepato-splanchnic bed, which likely reflects hepatic uptake, during exercise [39]. We measured a more than five-fold increase in the hepato-splanchnic flux of succinate during exercise, demonstrating the possibility of an endocrine action of succinate on hepatic cells (Figure 2).

**Figure 3:**
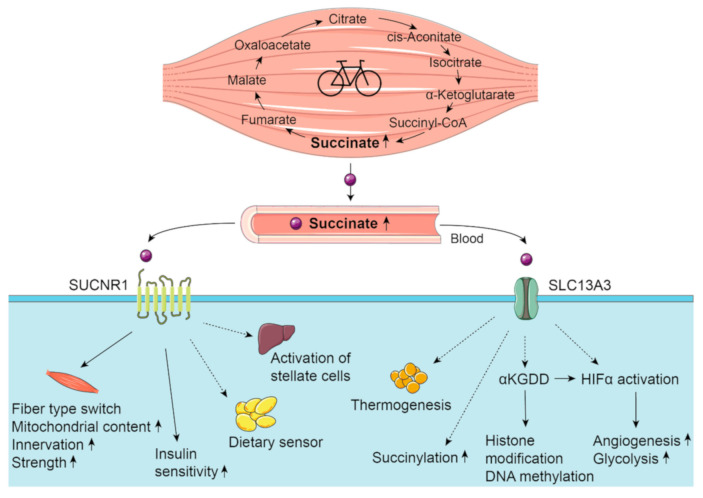
**Action of succinate as a myometabokine.** Skeletal muscle releases succinate during exercise, resulting in elevated plasma levels. Succinate then mediates signaling on target organs by either binding to its specific cell surface receptor SUCNR1 or after cellular uptake via SLC13A3. Solid arrows: succinate-mediated effect in exercise adaptation has been shown; dashed arrows: signaling function not studied in the context of exercise to date. The figure was created using Servier Medical Art templates, which are licensed under a Creative Commons Attribution 3.0 Unported License; https://smart.servier.com (accessed on 22 June 2021).

Succinate is one of the best-studied TCA cycle metabolites with regard to its signaling properties. In general, there are two mechanisms by which succinate exerts its intracellular signaling function: either by binding to its specific cell surface receptor, succinate receptor 1 (SUCNR1) alias G-protein coupled receptor 91 (GPR91), or after intracellular uptake, mainly via the sodium-dicarboxylate cotransporter SLC13A3 (Figure 3). SUCNR1 is a G_i/o_- or G_q_-coupled receptor [68] and succinate, but no other intermediate of the TCA cycle is able to activate the receptor, with EC_50_ values of 30–50 µM being in the same range as the physiological plasma concentrations during exercise [68]. SUCNR1 is expressed in many tissues (e.g., liver, kidney, heart), albeit with a cell-type-specific expression pattern. Thus, stellate cells likely account for most of its expression in the liver [69] and non-myofibrillar desmin-negative cells, such as endothelial, stromal and satellite cells, account for its expression in skeletal muscle [37]. While SUCNR1 was not detected in fused skeletal muscle cells or the myofibers of mice, controversy exists regarding its expression in the murine C2C12 skeletal muscle cell line [37,70]. Inspired by the discovery that long-term treatment with succinate leads to cardiac hypertrophy [71], Wang et al. [70] studied the potential of succinate to influence skeletal muscle morphology and function. They observed in mice that succinate causes a SUCNR1-mediated activation of the calcium signaling pathway and of the downstream transcription factors nuclear factor of activated T-cells (NFAT) and myocyte-specific enhancer factor 2 (MEF2). This induces a muscle fiber type switch from fast- to slow-twitch fibers, along with an increase in mitochondrial content. The recent work of Reddy et al. [37] suggests that succinate can function as a bioenergetic sensor released from the exercising myofibers and acting in a paracrine manner on the non-myofibrillar cells to initiate neurotrophic and muscle extracellular matrix remodeling. As a consequence, mice lacking SUCNR1 had less of an increase in muscle innervation and muscle strength after training [37]. In humans, the exercise-induced succinate increase correlated with the improvement in insulin sensitivity [37]. Thus, there is good evidence that succinate plays an important role as a myometabokine in the adaptation of skeletal muscle to exercise training.

SUCNR1-mediated signaling has been linked to a variety of other effects in different tissues. It modulates the renin-angiotensin system [68], leads to the activation of hepatic stellate cells, inducing a pro-fibrotic response [69,72], and stimulates osteoclastogenesis [73]. Furthermore, a function as a dietary sensor in adipose tissue has been described [74]. The relevance of these non-muscular succinate effects, mediated via activation of the succinate receptor, needs to be carefully considered in the context of exercise, as they were mainly reported in pathophysiological conditions, when succinate levels are chronically elevated.

The dicarboxylate transporter SLC13A3 has relevant expression levels in hepatocytes, renal proximal tubular cells, brain, and pancreas, but not in the skeletal muscle [45]. The co-transport of three Na^+^ and one succinate molecule is pH-dependent (optimum ~ pH 7.5–8.5) [45]. The K_m_ value for succinate cotransport was determined to be ~20 µM [75], which is in the same range as physiological plasma concentrations. An important physiological function of the cellular uptake of metabolites via SLC13A3 is the constant replenishment of the TCA cycle for mitochondrial respiration and gluconeogenesis [45].

In the cytosol, succinate can be a source of succinyl moieties for posttranslational lysine succinylation and thereby modify the function of proteins [76,77]. Zhang et al. [76] suggested that the impact of succinylation on protein structure and function is high compared to other common posttranslational modifications such as acetylation or methylation as the structural moiety is larger and replaces the positive charge from lysine with a net negative charge. Analysis of the mammalian succinylome demonstrated its potential impact on enzymes of the mitochondrial metabolism, including the TCA cycle [78]. Succinylation can be efficiently reversed by the protein sirtuin 5 (SIRT5) [79]. Studies in SIRT5 knockout mice pointed towards an essential interplay between protein succinylation and SIRT5 activity for cellular function and metabolic regulation [80,81]. Whether the increased release of succinate from the contracting skeletal muscle during exercise can lead to enhanced levels of protein succinylation has, to our knowledge, not been addressed to date.

An accumulation of succinate in the cytosol due to dysfunctional mitochondrial succinate dehydrogenase (SDH) was observed in tumor cells, and results in product inhibition of aKGDDs. HIF1α-prolyl hydroxylases (PHDs) belong to this group and are important for the degradation of HIF1α [82,83]. As a consequence of the inhibition of PHDs, HIF1α is stabilized [82,83], resulting in the expression of target genes involved in angiogenesis and glycolysis. In primary hepatocytes, succinate-driven HIF activation was related to increased levels of cAMP and an enhanced hepatic glucagon response [84]. A transcriptome analysis of livers of exercising mice pointed to HIF1α and cAMP as positive regulators of the acute transcriptional response to endurance exercise [39]. Additionally, the transcription factors cyclic AMP-responsive element-binding protein (CREB), cAMP-responsive element modulator (CREM) and forkhead box protein (FOXO), which are known to be regulated by cAMP in the liver, were also predicted to be activated [39]. Thus, skeletal muscle-derived succinate may promote hepatic adaptation to exercise by supporting cAMP-dependent gene activation.

Additional cytosolic aKGDDs that are inhibited by succinate include collagen prolyl-4-hydroxylases, the ten eleven translocation (TET) family of 5mC hydroxylases, and the α-KG-dependent Jumonji-C-domain-containing histone demethylases [18,85,86]. In vivo studies in the liver of mice provided evidence that accumulation of succinate caused by transient knockdown of SDH regulates histone and DNA demethylation [85]. Changes in mRNA expression after a single bout of exercise are associated with transient DNA hypomethylation in skeletal muscle [87]. Hence, succinate signaling at the epigenetic level by histone modification and DNA methylation can also be considered as a potential mechanism linking the exercise-induced increase in local and systemic succinate levels with changes in mRNA expression and, thus, exercise adaptation.

Succinate also exerts effects on adipose tissue. The oral supplementation of succinate in mice increases systemic succinate levels and whole-body energy expenditure, and reduces lipid accumulation in brown, subcutaneous and epididymal adipose depots and in liver [88]. The data were obtained during high-fat feeding, but the intravenous injection of succinate acutely increased whole-body oxygen consumption in chow-fed mice, supporting biological effects in normal-weight rodents without metabolic disturbances. The rapid metabolization of succinate by brown adipocytes provides a mechanism for the induced thermogenesis and energy expenditure, which may also be involved in the reported browning of adipocytes in trained mice [89].

## 6. Fumarate

Physiological fumarate blood concentrations in healthy humans are in the low micromolar range [40]. After endurance exercise, the plasma levels increase up to 2.5-fold [38,41] and remain elevated after 60 min of recovery [38]. During cycling at 70% VO_2_max, intramuscular concentrations of fumarate reached a seven-fold increase after 10 min [27]. At the end of the 95 min cycling test, fumarate was still elevated four-fold compared to pre-exercise levels. These results are supported by cycling tests performed at similar intensities (60–75% VO_2_max), where fumarate was quantified in muscle biopsies taken during and immediately after the exercise bout [25,43]. The comparison of fumarate obtained in blood samples collected from the pulmonary artery, reflecting exercising lower extremities, and from the superior vena cava during cardiopulmonary exercise testing revealed higher levels in pulmonary artery samples, suggesting a release of fumarate from the contracting skeletal muscle [38] (Figure 2).

The sodium-dependent cotransport via SLC13 proteins is suggested as the main route for the cellular uptake of fumarate. SLC13A2 and SLC13A5 have been shown to interact with and transport fumarate [45]. No specific receptor for fumarate has been discovered to date, to our knowledge. In contrast, the hydroxy-carboxylic acid receptor HCA2 binds fumarate esters and mediates the anti-neuroinflammatory effects of the pharmacological metabolite monomethylfumarate [90].

An accumulation of fumarate drives a chemical modification known as succination [91]. A michael addition between the fumarate and thiol groups of proteins leads to the formation of S-(2-succinyl)cysteine. Muscular glyceraldehyde 3-phosphate dehydrogenase (GAPDH) was the first enzyme described to be succinated [92], and the modification was accompanied by decreased enzymatic activity [93]. Succinated GAPDH was found in diabetic rats and suggested to be one mechanism of glucotoxicity [93,94]. Other proteins reported to undergo succination are mitochondrial aconitase 2 (ACO2) [95] and the Kelch-like ECH-associated protein 1 (KEAP1) [96]. KEAP1 forms a complex with nuclear factor erythroid 2-related factor 2 (NRF2), which, after KEAP1 succinylation is released, enables the nuclear translocation and activation of NRF2 target genes. Notably, the transcriptional activity of both NRF1 and NRF2 has been implicated in the adaptive response to endurance training [97], and a transcriptome analysis of the liver of exercising mice suggested elevated NRF2 activation [39]. NRF2 plays a central role in activating genes relevant for antioxidative defense and, together with peroxisome proliferator-activated receptor-gamma coactivator 1α (PGC1α), for mitochondrial biogenesis [98]. Fumarate has also been shown to be a potent competitive inhibitor of aKGDDs, based on its structural analogy to α-ketoglutarate.

Taken together, exercise-induced increases in fumarate concentration could influence enzyme activities, peptides and proteins and transcription factors through succination, and thereby exert a signaling role in exercise adaptation.

## 7. Malate

The human blood concentrations of malate under physiological conditions reach up to 20 µM [40]. Exercise of varying intensity and duration leads to increases in plasma levels in a range from 1.2- to 5.5-fold [38,41,99,100] that are sustained after a 60 min recovery period [38]. Intramuscular malate concentrations in the contracting skeletal muscle rise rapidly, reaching a peak of 6.5-fold of the resting value after 10 min, and remain elevated while exercise is continued [27]. Further studies measured up to nine-fold increases in intramuscular malate concentrations immediately after an acute bout of exercise [23,25,43]. In line with the increase in intramuscular and plasma concentrations, muscle interstitial malate increased 1.3-fold immediately after an acute bout of exercise [34]. Additionally, we provided evidence for malate being taken up into the liver during exercise by investigating the arterio-hepatovenous difference [39] (Figure 2). Cellular uptake of malate is suggested to be mainly mediated by SLC13A2 and SLC13A5 [45,46].

Based on the structural relationship with other TCA intermediates, it can be assumed that malate also influences cellular signaling cascades and epigenetic modifications. However, no function as a signaling molecule has been reported to date.

## 8. Concluding Remarks

TCA intermediates are important signals of the substrate oxidation and interconversion that take place in the exercising muscle. As metabolites shown to exert regulatory and signaling function, they fit into the concept of myometabokines. Most of their signaling effects have been studied in the context of pathophysiological changes in animal models, when TCA intermediate levels in plasma or tissue are chronically elevated or when cellular uptake is blocked by genetic inactivation of their transporters. To date, the best evidence for a regulation of physiological responses of the skeletal muscle and other organs in the context of exercise exists for succinate. Further studies are needed to reveal the signaling function of exercise-induced acute and transient changes in TCA metabolite levels in humans. This will help to gain a deeper understanding of how metabolites regulate exercise adaptation processes in skeletal muscle and other organs, and how they contribute to the beneficial effects on disease prevention and treatment.

## Figures and Tables

**Table 1:** TCA intermediates released by human skeletal muscle during endurance exercise.

Metabolite	Subjects	Type of Exercise	Exercise Protocol	Timepoints of Detected Release	Analytical Method	Reference
**Citrate**	*n* = 11 (m), 24 (21–28) years, healthy	one-leggedleg extension	80% W_max_ 60 min	10 min ^b^ 10 min after completion of exercise ^b^	femoral A-V diff., enzymatic	Hargreaves et al., 1991 [35]
	*n* = 6 (f + m), 24 (20–31) years, physically untrained, healthy	supine cycling	60–70% W_max_ 30 min	20 min 30 min	femoral A-V diff., enzymatic	Nielsen & Thomsen, 1979 [36]
	*n* = 5 (f + m), 21–46 years, healthy, BMI 18–35	upright cycling	65% HR_max_	30 min	interstitial fluid microdialysis, GC-MS	Zhang et al., 2019 [34]
**Isocitrate**	*n* = 5 (f + m), 21–46 years, healthy, BMI 18–35	upright cycling	65% HR_max_	30 min	interstitial fluid microdialysis, GC-MS	Zhang et al., 2019 [34]
**Succinate**	*n* = 10 (m), 27 ± 1 years ^a^, moderately physically active, healthy, BMI 24 ± 1 ^a^	upright cycling	67% VO_2max_ 60 min	60 min	femoral A-V diff., LC-MS	Reddy et al., 2020 [37]
	*n* = 8 (f + m), 48 ± 5 years ^a^, healthy, BMI 27 ± 1 ^a^	upright cycling	incremental ramp protocol	peak exercise	A-V diff. ^d^, LC-MS	Lewis et al., 2010 [38]
	*n* = 5 (f + m), 21–46 years, healthy, BMI 18–35	upright cycling	65% HR_max_	30 min	interstitial fluid microdialysis, GC-MS	Zhang et al., 2019 [34]
**Fumarate**	*n* = 8 (f + m), 48 ± 5 years ^a^, healthy, BMI 27 ± 1 ^a^	upright cycling	incremental ramp protocol	peak exercise	A-V diff. ^d^, LC-MS	Lewis et al., 2010 [38]
**Malate**	*n* = 8 (f + m), 48 ± 5 years ^a^, healthy, BMI 27 ± 1 ^a^	upright cycling	incremental ramp protocol	peak exercise	A-V diff. ^d^, LC-MS	Lewis et al., 2010 [38]
	*n* = 9 (m), 21 ± 1 years ^a^, healthy, BMI 23 ± 1 ^a^	one-leggedleg extension	50% W_max_ 120 min	60 min	femoral A-V diff., CE-MS	Hu et al., 2020 [39]
	*n* = 5 (f + m), 21–46 years, healthy, BMI 18–35	upright cycling	65% HR_max_	30 min ^c^	interstitial fluid microdialysis, GC-MS	Zhang et al., 2019 [34]

W_max_: maximal workload; VO_2max_: maximal oxygen consumption; HR_max_: individual maximal heart rate; n: number of subjects participating in the study; f: female; m: male; BMI: body mass index [kg/m^2^]; A-V diff.: arterio-venous difference over the exercising leg; GC: gas chromatography; LC: liquid chromatography; CE: capillary electrophoresis; MS: mass spectrometry. ^a^ mean ± SEM. ^b^ ~2-fold increased release compared to pre-exercise, no statistical significance calculated. ^c^ Dialysate collected during 30 min, total duration of exercise not reported, no statistical significance calculated. ^d^ Suggested muscular release (measurement of A-V diff. between pulmonary arteria and superior vena cava, representing total lower exercising body).
